# Exploring the feasibility of an artificial intelligence based clinical decision support system for cutaneous melanoma detection in primary care – a mixed method study

**DOI:** 10.1080/02813432.2023.2283190

**Published:** 2024-02-07

**Authors:** Jonatan Helenason, Christoffer Ekström, Magnus Falk, Panagiotis Papachristou

**Affiliations:** a AI Medical Technology, Stockholm, Sweden; b Department of Health, Medicine and Caring Sciences, Linköping University, Linköping, Sweden; c Department of Neurobiology, Care Sciences and Society, Karolinska Institutet, Stockholm, Sweden

**Keywords:** Artificial Intelligence, clinical decision support system, Cutaneous Melanoma, mobile health, primary care physicians

## Abstract

**Objective:** Skin examination to detect cutaneous melanomas is commonly performed in primary care. In recent years, clinical decision support systems (CDSS) based on artificial intelligence (AI) have been introduced within several diagnostic fields.

**Setting:** This study employs a variety of qualitative and quantitative methodologies to investigate the feasibility of an AI-based CDSS to detect cutaneous melanoma in primary care.

**Subjects and Design:** Fifteen primary care physicians (PCPs) underwent near-live simulations using the CDSS on a simulated patient, and subsequent individual semi-structured interviews were explored with a hybrid thematic analysis approach. Additionally, twenty-five PCPs performed a reader study (diagnostic assessment on the basis of image interpretation) of 18 dermoscopic images, both with and without help from AI, investigating the value of adding AI support to a PCPs decision. Perceived instrument usability was rated on the System Usability Scale (SUS).

**Results:** From the interviews, the importance of trust in the CDSS emerged as a central concern. Scientific evidence supporting sufficient diagnostic accuracy of the CDSS was expressed as an important factor that could increase trust. Access to AI decision support when evaluating dermoscopic images proved valuable as it formally increased the physician’s diagnostic accuracy. A mean SUS score of 84.8, corresponding to ‘good’ usability, was measured.

**Conclusion:** AI-based CDSS might play an important future role in cutaneous melanoma diagnostics, provided sufficient evidence of diagnostic accuracy and usability supporting its trustworthiness among the users.

## KEY POINTS

Effective primary care is important for discovering cutaneous melanoma, the deadliest and an increasingly prevalent form of skin cancer.

- ‘Trust’, ‘usability and user experience’, and ‘the clinical context’ are the qualitative themes that emerged from the qualitative analysis. These areas need to be considered for the successful adoption of AI assisted decision support tools by PCPs.
- The AI CDSS tool was rated by the PCPs at grade B (average 84.8) on the System Usability Scale (SUS), which is equivalent to ‘good’ usability.
- A reader study, (diagnostic assessment on the basis of image interpretation) with 25 PCPs rating dermoscopic images, showed increased value of adding an AI decision support to their clinical assessment.

## Introduction

Primary care and Primary Care Physicians (PCPs) play a vital role in managing patients with skin lesions of concern in order to detect cutaneous melanoma [1], in many parts of the world being the most rapidly increasing cancer form of all [2]. Melanoma detection in an early stage of the disease is associated with reduced risk of metastases, morbidity and mortality [3,4], as well as with lowered societal costs [2,5]. Given the severity of the disease, in terms of rising incidence and mortality rates and the associated costs, it is important to develop cost-effective methods to facilitate skin lesion examinations.

Today’s healthcare has undergone a digital transformation. New technologies such as Artificial Intelligence (AI), Telemedicine, and Smartphones have changed how patients interact with healthcare and how healthcare professionals redesign and improve healthcare related processes [6–8]. Areas such as medical image analysis have almost completely shifted to digital images [9]. The application of AI to these images has enabled physicians to perform faster and more accurate image interpretation [10]. While digitalization is an essential part of solving healthcare challenges, digitalization in itself is a challenge to implement [11,12].

Technological advances with mobile devices combined with health-related software applications have made it possible for mobile health (mHealth) devices to emerge, facilitating implementation in practice, with subsequent potential for improving healthcare systems and processes [13,14]. These technological advances have enabled opportunities for improving healthcare instruments such as clinical decision support systems (CDSS) [15]. AI has demonstrated its potential to help physicians to increase their diagnostic accuracy in several healthcare areas, including skin cancer detection, allowing them more efficient analysis of data [16–24]. However, AI-based systems have not been fully evaluated for their usability in primary care, nor for their acceptance by the practising physicians [1,18]. This study aims to investigate the feasibility of an AI-based CDSS for cutaneous melanoma, referred to as [the CDSS], in a primary care setting. A mixed method study was employed, with regard to PCPs user experience, instrument usability, perceived clinical applicability and value.

## Materials and methods

This study was designed as an exploratory and descriptive qualitative interview and quantitative reader study. The qualitative approach to the interview data was based on a hybrid thematic approach, combining inductive and deductive analysis [25–27]. The study was carried out between March and May 2021.

### The CDSS

The Dermalyser decision support system is developed as a platform-independent web application powered by AI and the experiments described were part of preliminary testing of the medical device software (MDSW) prototype (version 0.1). The AI used in the application was based on a convolutional neural network, trained on the photographic information of 6714 dermoscopic images of cutaneous melanomas and non-malignant pigmented skin lesions. The dermoscopic images were derived from the International Skin Imaging Collaboration (ISIC) 2020 challenge database [28]. Based on these, a diagnostic accuracy of 90% sensitivity and 65% specificity was reached in synthetic tests. The device is used by taking a dermoscopic photo of the skin lesion of concern, and uploading it to the AI, which returns a two-tailed clinical decision supportive statement, either as:” Melanoma cannot be excluded” or “No signs of Melanoma”.

### Near-live simulations with think aloud

To assess the usability, a near-live simulation was performed with fifteen voluntary primary care physicians at five different primary healthcare centres in Sweden [29–31]. During the simulation, a simulated patient scenario, intended to mimic a true doctor’s consultation with a patient seeking for a skin lesion of concern, was performed and video recorded. The scenario started with the simulated patient entering the doctor’s office, and the physician taking anamnesis and investigating the patient’s skin lesion following ordinary clinical routine. At an appropriate time chosen by the physician, the CDSS was applied to the process of diagnosing the skin lesion, which meant using the handheld dermatoscope connected to the mobile phone and following the instructions on the screen. During this part of the simulated consultation, the participants were asked to think aloud and express their thoughts and feelings about the device while using it. A test conductor was present in the room during the test, observing and giving instructions about the test procedure, but not interacting or giving instructions on how to use the CDSS. The near-live simulations were audio/video-recorded and what the physicians expressed verbally during its course written verbatim afterwards. In total, 19 PCPs were asked to participate. Four of the physicians asked to participate either declined or were unable due to their schedule and the time frame of the study. In the end, this study recruited 15 PCPs with varying experience and active at five different primary health care centres in Sweden (within Region Stockholm and Region Östergötland).

### Semi-structured interviews

After the PCPs had completed the near-live simulation, individual, semi-structured interviews were performed, addressing the experience of CDSS usefulness and its implementation in an everyday clinical workflow. The interviews took place in the respective healthcare centre after each near-live simulation and were also audio-video recorded for later transcription. The mean time of a conducted interview was 37 min. The full interview guide is presented in supplementary material (S1).

### Qualitative analysis with deductive-inductive approach

**Figure 1.**
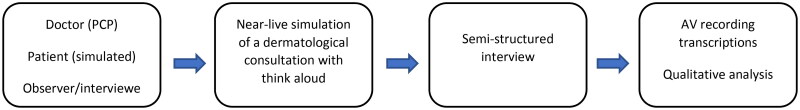
Flowchart of near-live simulations with think aloud, semi structured interviews and qualitative analysis.

The qualitative thematic analysis of the think aloud observations and semi-structured interviews was conducted through a combined deductive and inductive approach in three phases [25,27,32]. First, recordings were transcribed verbatim and data reviewed independently by two authors (J.H. & C.E.), where initial patterns were identified. Phase two, the deductive part of the approach, was conducted by combining these initial patterns with a set of predefined categories based on existing theory in the literature [27,33,34]. The authors then iteratively developed and applied initial codes to the transcribed data from phase one, while being guided in this process by the initial categories from phase two. In phase three, the authors conducted a data-driven inductive approach to identify themes and sub-themes emerging from the data from phase one and two [25,27]. All authors reviewed, named, and defined themes, and sub-themes according to an iterative process until consensus was reached. Representative quotes were selected to highlight themes and sub-themes [25]. The process from near-live simulations to interviews and qualitative analysis is described with a flowchart in Figure 1.

### System usability scale

Quantitative evaluation of perceived usability of the CDSS was measured on the System Usability Scale (SUS) [35]. The SUS consists of ten statements, odd-numbered statements being positively framed and evenly numbered statements negatively framed, to which the respondents choose the best-fitting of five response alternatives, scored 1-5 points (strongly disagree, disagree, neutral, agree, and strongly agree; supplementary material (S2)).

At SUS calculation, for each of the odd-numbered statements 1 point is subtracted from the response value, whereas for each of the even-numbered statements the response value is subtracted from 5. The generated scores are then added together into a summarised score and multiplied by 2.5, ending up in a range of possible values between 0 - 100. At interpretation, a score above 80 is generally considered to indicate high usability [36].

### Clinical assessment of AI tool

To assess the added value of an AI based CDSS, twenty-five PCPs performed a reader study (diagnostic assessment on the basis of image interpretation) in the form of an anonymous survey-based clinical assessment. Two sets of nine plus nine pre-diagnosed dermoscopic images of either cutaneous melanomas or benign skin lesions were presented in random order. Clinical responses were collected using an internet survey, presenting one image at a time. In the case of the first set of nine images, the participants gave their responses to whether they, based on their clinical evaluation of the lesion, would choose to 1) Perform an excision or refer the patient, 2) Send the patient home without follow-up, or 3) Other (free text field). In the second set of nine images, the clinicians were prompted with a recommendation from the AI based CDSS to either excise the lesion or send the patient home without follow-up before deciding on the above choice of three actions. Based on the clinicians’ responses sensitivity, specificity, positive predictive value (PPV), negative predictive value (NPV) and diagnostic accuracy were calculated.

### Ethical considerations

All participating primary care physicians received an email invitation to the study together with a detailed information sheet and informed consent. All participants provided their written informed consent prior to study participation. The present study followed the ethical principles for medical research of the Helsinki declaration [37]. There was no further requirement for ethical approval from the Swedish Ethical Review Authority, according to the Swedish Ethical Review Act [38].

## Results

### Qualitative analysis of near live simulations and semi-structured interviews

Analysis of the recorded near live simulations and the subsequent individual user interviews resulted in the generation of three themes (i.e. ‘Trust’, ‘Usability and User experience’, and ‘Clinical context’), together with ten sub-themes, in turn generated from 75 identified codes, as presented in Table 1. The consolidated criteria for reporting qualitative research (COREQ-32) were used in the description of the results (supplementary material S3) [39].

**Table 1.** Themes and subthemes generated from the qualitative analysis of the near-live simulations and individual interviews.

Theme	Sub-theme
Trust	The importance of trust
	Factors governing trust
Usability and User Experience	The Working Process
	Easiness to Use
	Integration with Electronic Health Record System
	Improvements and Additional Features
	Instructions and Education
Clinical Context	Responsibility and Relation to Guidelines
	Value for Clinical Practice
	Comparison With Ordinary Workflow

#### Trust

A prominent theme that was evident throughout all the interviews was *Trust*. Within this theme, two main aspects of trust were defined, namely *The importance of trus*t and *Factors governing trust*.

### The importance of trust

The importance of the healthcare system and the primary care physicians to feel that they can trust in a tool such as the CDSS, and its ability to provide reliable diagnostic guidance, was emphasised. The following quote describes how the tool is experienced to work well, but points out the need also to trust the tool:

I don’t know, it works well. But I hope I can trust it of course. (Respondent 5)

A concern that was expressed was that it might be hard to trust the tool enough to send a patient home with a lesion that the physician herself felt was suspicious, even if the tool determined it to be of low risk. This further highlight that a high degree of trust is important for a tool such as the CDSS to be adopted into the healthcare system and workflow:

If I’m thinking this looks a little suspicious then I would probably have a hard time, maybe just letting it go even if the phone says it’s benign. (Respondent 15)

### Factors governing trust

The other pre-dominant aspect of trust expressed by the respondents was when discussing different factors that were considered to govern the level of trust experienced towards a tool such as the CDSS. Among these, supportive medical evidence based on high-quality clinical trials evaluating the diagnostic accuracy of the CDSS’ was commonly seen, among the participants, as a key element for the tool to be trusted:

So, if a good clinical study would confirm that it works in practice, it would also make it easier for me to use the tool (Respondent 4)

Positive opinions from important actors within the dermatological field (key opinion leaders), such as dermatologists, were also thought to contribute to favouring trust in the CDSS.

Besides being certified, being recommended by dermatologists was also mentioned a factor that would facilitate trust in the tool:

And then, as we mentioned before, that it was certified and something that the dermatologists think you should use this, this is good (Respondent 9)

#### Usability and user experience

*This theme* highlights the important aspect of the CDSS’ clinical potential. Tapping into the previous theme, a well thought-through and designed interface was believed to contribute not only to a good user experience, but also to enhanced reliance in the product.

You have to feel that the user experience is good, and then that you can trust this. (Respondent 14)

### The working process

Conformity with *the working process* was described as central when using the CDSS. On the question to whether they felt that the CDSS in any way obstructed their working process, several of the respondents gave answers similar to the following quotes:

No, now it’s the first time, and then it takes some time to understand the technology. But I do not think there will be any problems. (Respondent 8)

It is very convenient that it is possible to get an answer immediately. Extremely practical and timesaving. (Respondent 10)

### Ease of use

All the participants gave positive feedback on the *ease of use* of the CDSS, in different ways. This positive feedback could often be attributed to specific properties of the CDSS, such as its fastness in delivering its response from the AI. However, it was also expressed by several of the respondents how much they appreciated the CDSS as a whole. The following quotes captures a common opinion among the respondents, regarding easiness to use:

And the app itself is fantastic, so you do not have to take as many pictures as with [the teledermoscopy system], but this went very fast. (Respondent 5)

It was quite intuitive. I mean because I have not used this, I did not know how to turn on the lamp and so on … But I mean you remember, so it was very easy to use. (Respondent 11)

A little more specifically, several of the respondents also mentioned the speed of the AI-generated response as a positive surprise, as exemplified by the following quote:

I was surprised that the answer came so quickly, I am used to thinking that it will take several weeks. (Respondent 4)

Several of the respondents expressed how they expected the CDSS to allow for pinch gesture style zoom-ability. In the current version of the CDSS any pinch gestures only lead to the browser hosting the application to zoom in.

*Yes, but then I see … Is it possible to enlarge? … (Respondent 11)*

During the near-live simulations, several of the physicians had minor initial problems with the physical dermoscopic module that was used during the test session. Specific problems with the dermoscopic module were explicitly expressed during the interviews:

The downside is that the phone spins, because it spins with the handle here now when you have to adjust the focus on it. (Respondent 15)

### Integration with electronic health record system

Working in digital patient record systems is a highly integrated feature of physicians’ everyday workflow, which was reflected in the interviews. A feature not yet implemented in the studied version of the DDSS was an integration with the patient record system. However, such a connection was perceived not to be necessary.

I do not think that this needs to be documented for the medical record system. But it would be good if it was possible to connect… But I mean, many more things are more important to connect with the journal system today. (Respondent 11)

Some of the respondents even argued that a connection to the medical journal systems risks being purely negative. Anyhow, if implemented as an integrated or connected part of the digital patient record system, such connection would need to be very intuitive.

Maybe if it was very intuitive or smooth and I got good instructions… (Respondent 14)

### Improvements and additional features

There was no obvious consensus among the physicians regarding potential improvements that might enhance the CDSS’ clinical usefulness. One respondent reflected on the importance of practitioners getting some educational feedback of some kind in using the CDSS:

Everyone must expect them to receive some form of education, that this is how it works. (Respondent 14)

The value that the ability to diagnose more types of skin conditions using the DDSS would add to the applicability in clinical practice was expressed:

If it’s just malignant melanoma, it’s perfect. But, [I] would like to see that it could analyse squamous cell carcinoma and basal cell carcinoma. (Respondent 5)

The absence of other types of information than solely the dermoscopic image, compared to when using ordinary teledermatology was described as an important difference, in both negative and positive terms. Whereas the known impact of relevant anamnestic information, such as how the skin lesion had evolved over time, can be weighed in when consulting by teledermatology, this also puts higher demands on the physician to be able to provide such information, taking somewhat more time in demand.

This one has only evaluated the pictures. Seen from that aspect, it will be a narrower assessment as well. As with [the teledermatology system] or so, when you send them there, there is a little anamnesis, a little what symptoms they had, how fast was the change, previously removed, etc. So, it takes more of my decision-making processes into the assessment. (Respondent 9)

Additionally, choosing the right hardware with easy handling might improve the outcome.

It was a bit bumpy to set the focus, it makes the camera move… I have to say… (respondent 12)

### Instructions and education

Adding instructions for how to use the CDSS, to make usage even more apparent to the user, could, as expressed by the participants, be done by giving the user a short intro when the application is started or through a more guided usage process:

I think it is user friendly, just that you would get a little intro here. A couple of minutes, that this is how it works. Then it is very smooth and not a lot of hassle and extra icons, slim, quite easy to navigate and clear … It only says one word, camera, diagnosis, you might have wished you understood step by step that okay, now you should take a picture first and then… A little more like a flow almost. Here you get a menu, you get it… But maybe more like a ow, that this first… One, two, three, maybe so. (Respondent 14)

Although most of the respondents described the CDSS as intuitive potential usability improvements were also suggested, as demonstrated by the following quote:

It was probably more that I did not technically understand how the system works, and thought that there would be more instruction in the system. (Respondent 6)

#### Clinical context

The last generated theme identified different aspects of how the use of the CDSS conformed with the clinical context, further reflected in the following three sub-themes.

### Responsibility and relation to guidelines

A recurrent concern raised around the implementation of the CDSS emerged from the question who would actually be viewed as responsible for the medical decision in case the CDSS ruled out a lesion as benign which later on showed to be malignant (the doctor or the system), emphasising the importance of profound scientific data on its performance.

It would take quite a lot of evidence that this is accurate. The question is what happens if I free someone from a melanoma based on the tool, am I responsible for it or the person who developed this app? (Respondent 3)

The respondents expressed the significance of some kind of common policy or consensus on how to use the CDSS in the clinical work, and when, and that such consensus among colleagues, or adherence to guidelines, would support adoption in daily practice, as exemplified in the following quote:

Well…It has to do with what you, at the health centre, agree should be the routines actually…I guess…(Respondent 11)

### Value for clinical practice

The actual value of the CDSS for clinical practice was a central subject reflected upon among the participants. The fact that access to diagnostic supportive advice would be easily accessible by having it installed on a cell phone, was seen as beneficial, potentially saving time and resources. Instead of needing to look for clinical guidance or second opinion from a primary care colleague, or consulting a dermatologist, using the CCDSS could be a feasible and time saving alternative:

I might have benefited from this instead of going to get a colleague, I think. (Respondent 12)

Besides the rapid response, increasing the sense of security around the clinical evaluation of a skin lesion, by using the CDSS, was another variable described as positive, both for the physician, but also for the patient. When the physicians felt uncertain of their own judgement, the CDSS was perceived to contribute additional value to overbridge this uncertainty.

So, the big difference is that you get a rapid response and that I can feel more confident with my evaluation, and that the patient hopefully can feel a bit more confident too. (Respondent 4)

There were also some more cautious considerations expressed, stating that the reliance on the CDSS, and the extent of actual benefit is something that builds up from one’s own experiences of using it. In case of frequently presenting an outcome far from your own judgement, doubts about its reliability would be raised:

If there are many discrepancies, and major, between my evaluation and this [the CDSS], then maybe I won’t have that much use of it. (Respondent 8)

### Comparison with ordinary workflow

Teledermatology, by sending photos of the skin lesion in question, was the dominating experience the respondents mentioned having with other, already existing solutions aiming to facilitate and improve the diagnostic procedure of melanoma suspected skin lesions. In the semi-structured interviews, an explicit comparison with teledermatology services was expressed. While use of teledermatology was pointed out to provide more detailed feedback, the significantly greater speed at which the AI-generated feedback from the CDSS was received was seen as an advantage for both the physicians and the patients.

If we work with [the teledermatology system], it comes with a recommendation in the answer, along with a description. But this one [the CDSS] is simpler, and I am the one who has to make a decision, of course. (Respondent 5)

Especially in the case of patients with several skin lesions of concern, i.e. having a large number of pigmented nevi, or patients with previous cutaneous melanoma, the immediate response provided by the CDSS was considered an advantage compared to conventional teledermatology. Similarly, the need to take several pictures using teledermatology (compared to a single photo when using the CDSS), was described.

I am thinking above all of the patients who have had melanoma and will have an annual check-up, then there can be quite a few lesions to look at, and then it is a clear advantage, this direct feedback, versus teledermoscopy and keep on taking six pictures; it’s a damn job. (Respondent 9)

### System usability scale results

All 15 participating PCPs completed the SUS questionnaire, for which the results are presented in Supplementary Figure 1. As shown, the individual scores ranged between 72.5 (grade C), and 97.5 (grade A). The average score was 84.83 (SD ±7.22; grade B). According to Bangor et al. a SUS score above 72 is considered acceptable, and above 85 to be considered excellent [40].

### Clinical assessment of AI tool outcome

Table 2 shows the results of the survey based clinical assessment of dermoscopic images, both without and with the presented AI decision support tool information. Depending on the PCPs choice of predefined actions, sensitivity, specificity, PPV, NPV and diagnostic accuracy were calculated. As seen in Table 2, there was an increase in both sensitivity, specificity and diagnostic accuracy when following the same decision path as the AI-based CDSS.

**Table 2.** Results from the PCPs reader study (diagnostic assessment on the basis of image interpretation) without and with the use of AI. n = number of clinical assessments.

	Sensitivity (%) [95% CI]	Specificity (%) [95% CI]	PPV (%) [95% CI]	NPV (%) [95% CI]	Accuracy (%) [95% CI]
PCP + no AI (*n* = 189)	54.8 [38.7 − 70.2]	41.5 [33.4 − 49.9]	21.1 [13.9 − 30]	76.3 [65.4 − 85]	44.4 [37.2 − 51.8]
PCP + AI (*n* = 200)	91.1 [78.8 − 97.5]	48.4 [40.3 − 56.5]	33.9 [25.5 − 43.1]	94.9 [87.5 − 98.6]	58.0 [50.8 − 64.9]

## Discussion

This study investigated PCPs perceptions of the feasibility to use an AI-based CDSS application for cutaneous melanoma detection, in a typically primary care clinical context. The qualitative analysis concluded three themes ‘Trust’, ‘Usability and User experience’, and ‘Clinical context’ (alongside with ten subthemes). The results indicated that the CDSS was overall perceived as easy to use and understand, and that it contributed with important advantages such as quick response on recommendation based on the AI processing. The importance of trust is a big prerequisite in order to rely on it as a tool to be implemented in clinical routine. This further depends on the AI based CDSS being thoroughly investigated in a relevant clinical trial, assessing its diagnostic accuracy.

### Findings in relation to other studies

Concerns about the integration of AI based clinical decision support tools with ordinary workflow have been raised in some previous studies, and its possible effect on the doctor–patient interaction and patient-centredness of care [7,41,42]. Lim et al. found that patients in general prefer to have their skin lesions examined by a doctor, and not only by an AI device, pointing out the interactivity with the doctor as important [43]. This supports the opinion that AI in medicine should be used in combination, or under the supervision of human medical expertise [44]. However, these concerns were, in the same studies, also balanced by potential benefits thought to gain both patients and physicians, by means of facilitating the working process, saving time and effort, and contributing with valuable information [7,41,44]. This is in concordance with what was expressed by the respondents in the present interview study, the time-saving potential of the CDSS being highlighted as an important aspect in this regard. A crucial factor for time efficiency of a technical device of any kind is ease of use, especially when experiencing shortage of time [45] (which is often the case in daily practice in primary care), as illustrated in the results.

Another important concern associated with AI in medicine is the person that is medically responsible for the advice produced by the CDSS. One can make arguments for this being the manufacturer, the user or even the CDSS [46]. This was thought about by the respondents in the present study and is likely to be an important issue that will arise when integrating AI tools for diagnostic purposes. However, no diagnostic method has perfect accuracy, and this issue is present for any diagnostic methodology that is associated with some degree of uncertainty. According to the respondents, if the CDSS suggests a lesion to benign, but the physician thinks that it looks suspicious, the choice of action would still be to treat it according to one’s own evaluation, as also previously reported [45].

The quantitative results of this study indicate an increase in “doctors’ performance” when following the AI CDSS tool information, demonstrating the increased clinical value of having an additional decision support to consult. Results from previous studies indicate that AI can perform better than physicians in the diagnosis of cutaneous melanoma [16–18], which agrees with the outcome of the clinical assessment of the AI tool of our study, at least when performed in a simulated situation. In this regard, it is interesting to note that the PCPs increased both their sensitivity and level of diagnostic accuracy by using the CDSS, and at the same time just as interesting that they to a relatively high degree decided to hold on to their own initial decision, instead of relying on the CDSS guidance. A reasonable explanation for the latter finding is that a considerable degree of trust and confidence in any diagnostic supportive tool is needed to dare to rely on it, and to overrule one’s personal clinical experience. This is *per se* supported by the results in the qualitative part of the study, the importance of trust standing out as a crucial element for the clinical applicability as well as prerequisites to be further implemented in future clinical practice. The sources for generating such trust were in the study expressed either in terms of a clinical investigation supporting the diagnostic accuracy of the tool, or dermatology specialist colleagues endorsing it and recommending its usage in clinical practise.

### Strengths and limitations

The study has some limitations to be mentioned. One of these is the use of simulated patient situations in the evaluation of instrument usability, naturally not fully equalling real life patient consultations. However, the method allows for thorough and systematic observation of the actual thoughts, considerations and reflections made while using the CDSS, both by visual observation and through the think aloud recordings, unrestrained by stressful time limits in the clinic or diagnostic concerns [29,30]. Regarding the quantitative study part, lack of sample size and power calculation is a limitation, as well as the fact that there were two different sets of dermoscopic images (two melanomas in each set) that impair proper comparison of diagnostic accuracy of the AI tool. Looking at usability, participants were surprisingly consistent in their SUS scoring, and in all 15 individual cases reporting a value above the “acceptable” level [40].

To date, although found to be high and tested on a large sample, the diagnostic accuracy of the CDSS in discriminating cutaneous melanoma from benign pigmented lesions, has only been established through in-silico testing of the model against a database of unseen images. This should provide a good estimate for the real-world performance of the CDSS but the performance of an algorithm in a computer is never exactly the same as the performance in the real world. Hence, a prospective clinical investigation in a true population of patients seeking primary care for examinations of skin lesions of concern, would be necessary to both establish the diagnostic accuracy of the CDSS in this population, as well as its clinical value.

Overall, the perceptions about the CDSS, and its potential use in clinical practice were promising. In conclusion, AI-based CDSS might play an important future role in cutaneous melanoma diagnostics, provided that sufficient evidence of diagnostic accuracy and usability can be presented, adding up to increased trust and clinical value for both clinicians and patients.

## Supplementary Material

Supplemental Material

Supplemental Material

Supplemental Material

Supplemental Material
