# Supplementary figures and images for: Exploring the feasibility of an artificial intelligence based clinical decision support system for cutaneous melanoma detection in primary care – a mixed method study
Source: Scand J Prim Health Care. 2024 Feb 7;42(1):51–60. doi: 10.1080/02813432.2023.2283190 (PMC10851794; doi:10.1080/02813432.2023.2283190)

**
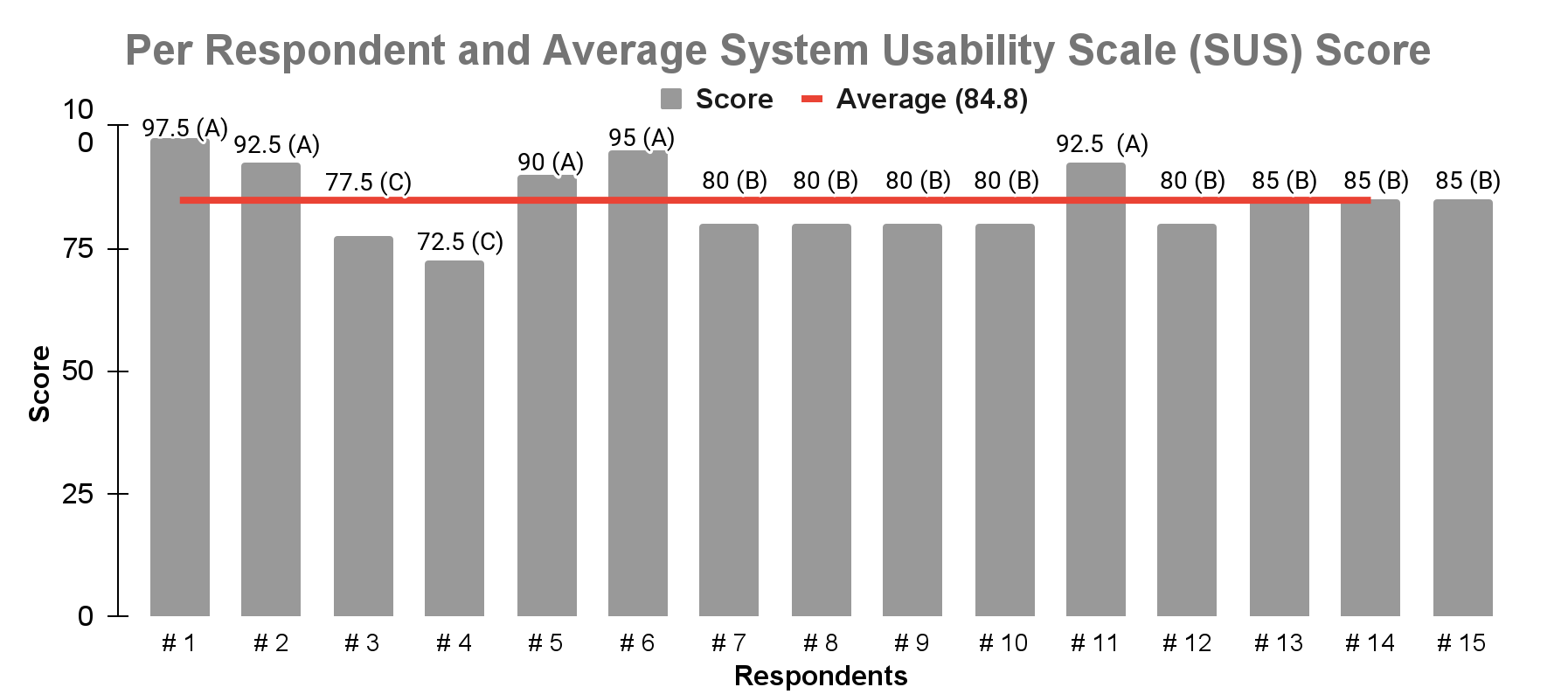
**

Supplement: Supplemental Material [file IPRI_A_2283190_SM7771.docx]
