# Supplementary material for: Exploring the feasibility of an artificial intelligence based clinical decision support system for cutaneous melanoma detection in primary care – a mixed method study
Source: Scand J Prim Health Care. 2024 Feb 7;42(1):51–60. doi: 10.1080/02813432.2023.2283190 (PMC10851794; doi:10.1080/02813432.2023.2283190)
Supplement: Supplemental Material [file IPRI_A_2283190_SM7753.docx]

## S3. Table_COREQ-32 checklist

**How the study followed the 32 criteria for reporting qualitative research according to a checklist presented by Tong et al.**

| **Domain (REF)** | **Criteria (REF)** | **Study criteria fulfilment** |
| --- | --- | --- |
| About the research team | 1. Interviewer | The lead author (J.H) |
|  | 2. The author’s credentials | Lead author (J.H.): M.Sc.  Second author (C.E.): M.Sc.  Third author (M.F.): MD, Ph.D.  Fourth author (P.P): MD. Ph.D. |
|  | 3. The authors’s occupation | Current occupation of lead author (J.H.) is lead software developer at AI Medical Technology. Current occupation of the second author (C.E.) is CEO at AI Medical Technology. Current occupation of the third author (M.F.) is ass. Professor at LiU and specialist physician/researcher at Region Ostergotland. Current occupation of the fourth author (P.P.) is researcher at KI and specialist physician/researcher at Region Stockholm. |
|  | 4. The author’s gender | All authors are males |
|  | 5. The author’s experience and training | The lead author (J.H.) has a BSc in computer science and holds a MSc in immersive technologies from Stockholm University in Stockholm, Sweden.  The second author (C.E.) has a BSc in computer science and holds a MSc in immersive technologies from Stockholm University in Stockholm, Sweden.  The third author (M.F.) is a specialist in General Medicine and an experienced researcher and ass. Professor. He holds a Ph.D. in medicine from the Linköpings University in Linköping, Sweden.  The fourth author (PP) is a specialist in General Medicine and an experienced researcher. He holds a Ph.D. in medicine from the Karolinska Institutet in Stockholm, Sweden. |
|  | 6. The author’s relationship with the participants | The lead author (J.H.) conducting the interviews had no relationship with the participants before the interview. The objective was to create a situation where the interviewees felt comfortable during their interview. |
|  | 7. The participants’ knowledge of the interviewer | All potential interview candidates received an email invitation together with a detailed participant information sheet about the study scope and objectives. No information was provided to the participants about the lead author except she had the role of being a medical student. |
|  | 8. Interviewer characteristics | The interviewer (lead author) has prior experience from IT, management, and takes a cross-scientific perspective (healthcare, IT, management). |
| design | 9. Methodological orientation and theory | Thematic analysis |
|  | 10. Sampling | Purposive |
|  | 11. Method of approach | Physical meetings |
|  | 12. Sample size | 15 |
|  | 13. Non-participation | 19 (estimated – some stakeholders asked colleagues about their interest to participate) |
|  | 14. Setting of data collection | At interviewee’s workplace |
|  | 15. Presence of non-participants | None |
|  | 16. Description of sample | Participants from both public and private primary healthcare organisations were invited with a purposive sampling. The selected primary care centres were active in Region Stockholm or Region Östergötland. |

|  | 17. Interview guide | The questions were developed by the authors, not piloted before, but confirmed and refined after initial pilot interviews. |
| --- | --- | --- |
|  | 18. Repeat interviews | n/a |
|  | 19. Audio/visual recording | Audio and visual recording |
|  | 20. Field notes | n/a |
|  | 21. Duration | Avg. 43 minutes per interview. |
|  | 22. Data saturation | Data saturation was discussed in the report. During the final interviews, the response-patterns started to repeat. |
|  | 23. Transcripts returned | No |
| Analysis and findings | 24. Number of data coders | Data was initially transcribed by J.H and C.E., coded by J.H. and later validated, in several iterations, together with M.F. and P.P. |
|  | 25. Description of coding tree | Example provided in Appendix. |
|  | 26. Derivation of themes | Theme and sub-theme were derived from the data. |
|  | 27. Software used to manage the data | Excel document, stored on secure cloud service |
|  | 28. Participant checking reporting | The participants did not provide feedback on the findings. |
|  | 29. Quotations presented | Yes, participant quotations were presented to illustrate the thematic analysis. |
|  | 30. Data and findings consistent | There was a consistency between data presented and the findings. |
|  | 31. Clarity of major themes | Themes and sub-themes were presented in the findings. |
|  | 32. Clarity of minor themes | Some minor themes (mentioned by a minority) were presented. |

## 
