# Supplementary material for: Exploring the feasibility of an artificial intelligence based clinical decision support system for cutaneous melanoma detection in primary care – a mixed method study
Source: Scand J Prim Health Care. 2024 Feb 7;42(1):51–60. doi: 10.1080/02813432.2023.2283190 (PMC10851794; doi:10.1080/02813432.2023.2283190)
Supplement: Supplemental Material [file IPRI_A_2283190_SM7752.docx]

## S2. Table_System Usability Scale statements

Statements in the System Usability Scale, each to be scored 1-5 (strongly disagree, disagree, neutral, agree, and strongly agree) by the respondent.

| 1.  2.  3.  4.  5.  6.  7.  8.  9.  10. | I think that I would like to use this system frequently.  I found the system unnecessarily complex.  I thought the system was easy to use.  I think that I would need the support of a technical person to be able to use this system.  I found the various functions in this system were well integrated.  I thought there was too much inconsistency in this system.  I would imagine that most people would learn to use this system very quickly.  I found the system very cumbersome to use.  I felt very confident using the system.  I needed to learn a lot of things before I could get going with this system. |
| --- | --- |
