# Supplementary material for: Exploring the feasibility of an artificial intelligence based clinical decision support system for cutaneous melanoma detection in primary care – a mixed method study
Source: Scand J Prim Health Care. 2024 Feb 7;42(1):51–60. doi: 10.1080/02813432.2023.2283190 (PMC10851794; doi:10.1080/02813432.2023.2283190)
Supplement: Supplemental Material [file IPRI_A_2283190_SM7713.docx]

## S1. Table_Interview guide

**Interview questions for stakeholders**. **English translation.**

| # | **Given the scenario you have just experienced:** |
| --- | --- |
| 1 | How would you describe your experience with patient cases like this? |
| 2 | If you were to try to sum up what you just experienced, how would you do it? |
| 3 | How would you compare this scenario to a “real” patient case? |
| 4 | What are your important decision-making processes in handling this patient case?   \| 4.1 \| Is there a lack of support for any of your decision-making processes in Dermalytics? \| \| --- \| --- \| |
| 5 | What would make you use the tool in your everyday work? |
| 6 | What would make you not use the tool in your everyday work?   \| 6.1 \| Not the journal? \| \| --- \| --- \| |
| 7 | Was it at any time during use that you felt that your work process was hindered? |
| 8 | How did you feel when you saw the decision support recommendation? |
| 9 | Was it ever during use when you experienced insecurity? |
| 10 | What do you think about the decision support as part of your clinical everyday life when assessing skin changes? |
| 11 | Is there any functionality or feature that you were missing in the tool?   \| 11.1 \| For example, connection to patient journal systems? \| \| --- \| --- \| |
| 12 | When you have used other decision support systems or digital tools within the context of your clinical everyday life, what are examples of features with these that have impressed you, or that you have experienced as positive?   \| 12.1 \| Can you mention any examples of features of the said tool that made you feel less inclined to use the tool in question? \| \| --- \| --- \| |

**Interview questions for stakeholders**. **Swedish version.**

| # | **Givet det scenario du precis har fått erfara:** |
| --- | --- |
| 1 | Hur skulle du beskriva din erfarenhet kring patientfall som det här? |
| 2 | Om du skulle försöka summera det du precis fick uppleva, hur skulle du göra det? |
| 3 | Hur skulle du jämföra detta scenario med ett “riktigt” patientfall? |
| 4 | Vilka är dina viktiga beslutsprocesser vid handläggande av det här patientfallet?   \| 4.1 \| Saknas stöd för några av dina beslutsprocesser i Dermalytics? \| \| --- \| --- \| |
| 5 | Vad skulle få dig att använda verktyget i din arbetsvardag? |
| 6 | Vad skulle få dig att inte använda verktyget i din arbetsvardag?   \| 6.1 \| Inte journalen? \| \| --- \| --- \| |
| 7 | Var det någon gång under användandet som du upplevde att din arbetsprocess blev förhindrad? |
| 8 | Vad fick du för känsla när du såg beslutsstödets rekommendation? |
| 9 | Var det någon gång under användandet då du upplevde osäkerhet? |
| 10 | Vad tänker du om beslutsstödet som en del i din kliniska vardag vid bedömning av hudförändringar? |
| 11 | Är det någon funktionalitet eller egenskap som du saknade i verktyget?   \| 11.1 \| Exempelvis koppling till journalsystem? \| \| --- \| --- \| |
| 12 | När du använt andra beslutsstöd eller digitala verktyg inom ramarna för din kliniska vardag, vad är exempel på egenskaper med dessa som imponerat på dig, eller som du upplevt som positiva?   \| 12.1 \| Vad är exempel på egenskaper hos nämnda verktyg som fått dig att känna dig mindre benägen att använda verktyget i fråga? \| \| --- \| --- \| |
